# *QuickStats:* Age-Adjusted Death Rates* from Female Breast Cancer,^†^ by State — National Vital Statistics System, United States,^§^ 2017

**DOI:** 10.15585/mmwr.mm6827a4

**Published:** 2019-07-12

**Figure:**
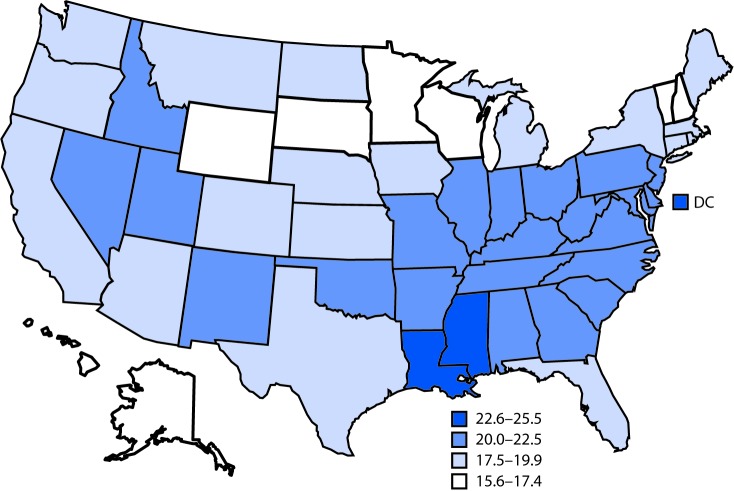
In 2017, the overall age-adjusted death rate for female breast cancer was 19.9 per 100,000 population. The highest death rates were in Mississippi (25.5), DC (24.3), and Louisiana (23.6). The lowest death rates were in Hawaii (15.6), Alaska (16.3), New Hampshire (16.3), Wyoming (16.5), Rhode Island (16.6), Minnesota (16.7), South Dakota (17.3), Wisconsin (17.4), and Vermont (17.4).

**Source:** National Center for Health Statistics, National Vital Statistics System, mortality file. https://www.cdc.gov/nchs/nvss/deaths.htm.

